# Spatial variability and source analysis of soil heavy metals: A case study of the key planting area of special agricultural products in Cangxi County, China

**DOI:** 10.1371/journal.pone.0303387

**Published:** 2024-05-10

**Authors:** Ziwei Feng, Wende Chen, Yichen Meng, Haixia Lu, Xinyi Shi, Jiajun Zhang

**Affiliations:** College of Geography and Planning, Chengdu University of Technology, Chengdu, China; Universidade de Vigo, SPAIN

## Abstract

Heavy metal pollution in farmland soil represents a considerable risk to ecosystems and human health, constituting a global concern. Focusing on a key area for the cultivation of special agricultural products in Cangxi County, we collected 228 surface soil samples. We analyzed the concentration, spatial distribution, and pollution levels of six heavy metals (Cr, Cu, Pb, Ni, Zn, and Hg) in the soil. Moreover, we investigated the sources and contribution rates of these heavy metals using Principal Component Analysis/Absolute Principal Component Scores (PCA/APCS) and Positive Matrix Factorization (PMF) models. Our findings indicate that none of the six metals exceeded the pollution thresholds for farmland soils. However, the mean concentrations of Cr and Ni surpassed the background levels of Sichuan Province. A moderate spatial correlation existed between Pb and Ni, attributable to both natural and anthropogenic factors, whereas Zn, Cu, Hg, and Cr displayed a strong spatial correlation, mainly due to natural factors. The spatial patterns of Cr, Cu, Zn, Pb, and Ni were similar, with higher concentrations in the northern and eastern regions and lower concentrations centrally. Hg’s spatial distribution differed, exhibiting a broader range of lower values. The single pollution index evaluation showed that Cr and Ni were low pollution, and the other elements were no pollution. The average value of comprehensive pollution index is 0.994, and the degree of pollution is close to light pollution. Predominantly, higher pollution levels in the northern and eastern regions, lower around reservoirs. The PCA/APCS model identified two main pollution sources: agricultural traffic mixed source (65.2%) and natural parent source (17.2%). The PMF model delineated three sources: agricultural activities (32.59%), transportation (30.64%), and natural parent sources (36.77%). Comparatively, the PMF model proved more accurate and reliable, yielding findings more aligned with the study area’s actual conditions.

## 1. Introduction

Soil is crucial for human survival, social development, food security, and health [1]. The rapid advancement of industrialization and urbanization has heightened the issue of soil heavy metal pollution worldwide. Significant levels of such pollution have been detected in various countries, including Bangladesh, Russia, Iran, and Saudi Arabia [2–5]. Given its status as the most populous developing nation, China, with its population exceeding 1.4 billion, also confronts this challenge. Pb, Cd, Zn, Ni, and As are the main pollutants in industrial heritage sites, while Cd is the main pollutant in agricultural soils, and the pollution of Cd and Hg is on the rise [6,7]. Under natural conditions, soil heavy metals are difficult to degrade and can accumulate easily. If ingested by humans through the food chain, these metals can lead to kidney failure, damage the central nervous system, increase cardiovascular disease risk, disrupt prostate homeostasis, and potentially cause various cancers [8,9]. Consequently, analyzing the spatial distribution and sources of soil heavy metals is crucial for preventing and controlling heavy metal pollution, thereby reducing its detrimental effects on human health and the environment.

The distribution of heavy metals in soil exhibits complexity and variability, both spatially and temporally, influenced by natural factors like climate, topography, and biological processes, as well as human activities [10]. Soil properties exhibit spatial dependence; according to the first law of geography, sample points closer to each other tend to have more similar properties than those farther apart. Classical statistics, which assume measurements are independent, fail to analyze this spatial dependence [11]. Geostatistics, the primary research method in the theory of spatial variability, employs the semi-variogram function and kriging interpolation to analyze the spatial correlation and dependence of natural phenomena [12]. First proposed by D.G. Krige, a South African geologist, in 1951, and subsequently developed and refined by the renowned French geologist Matheron [13], geostatistics has evolved into a valuable technique for quantifying soil parameter characteristics and conducting spatial interpolation [14].

The sources of heavy metal pollution can primarily be categorized into two types: natural sources and anthropogenic (human-induced) activities [15]. Typically, natural sources do not lead to serious pollution; instead, human activities are regarded as the primary contributors to soil heavy metal pollution [16]. These activities include coal mining, motor vehicle exhaust emissions, and the use of pesticides and fertilizers, along with fossil fuel combustion and sewage irrigation [15,17]. Source analysis plays a crucial role in identifying the origins of heavy metals and mitigating heavy metal pollution [18]. Commonly employed analytical methods include multivariate statistical models such as Principal Component Analysis (PCA), Correlation Analysis, Cluster Analysis (CA), Factor Analysis (FA), Positive Matrix Factorization (PMF), and the PCA/Absolute Principal Component Scores (PCA/APCS) receptor model [19]. Correlation Analysis, PCA, CA, and FA are traditional multivariate statistical methods capable of qualitatively identifying pollution sources. However, they fall short in quantifying each source’s contribution. In contrast, the PMF and PCA/APCS models offer a more quantitative approach in pinpointing potential sources of pollution. The Positive Matrix Factorization (PMF) model, grounded in elemental concentration matrices and associated uncertainties [20], was initially developed for source resolution in airborne particulate matter [21,22]. Its application has expanded significantly in recent years, encompassing the identification of heavy metal sources in soil, water sediments, and urban roads [23–25]. PCA/APCS, an exploratory tool that merges Principal Component Analysis with Multiple Linear Regression, investigates the structure of multivariate datasets [26]. It is also employed to estimate the contributions of individual contamination sources to specific species [27]. Both PMF and PCA/APCS models serve as straightforward and efficient tools for source resolution. To enhance the accuracy and reliability of their results, these models are often compared against each other. The optimal model varies depending on the specific subjects of study and geographic regions. For example, LiPing [28]et al. employed two models to pinpoint the sources of heavy metals in the subtropical agricultural soils of eastern China. Upon comparison, the PMF model was determined to be more suitable. Haji Gholizadeh Mohammad [29] et al. similarly employed two models to quantify the sources of pollution in three major rivers in South Florida. The results indicated that the PCA/APCS model is more suitable for the actual study area. The selection of the most appropriate model for investigating the sources of heavy metals can offer a scientific foundation for the prevention of pollution sources and the risk management of soil heavy metals.

In recent decades, numerous scholars have conducted extensive research on the spatial distribution and sources of heavy metals in soil, with most studies focusing on industrial, agricultural, and urban areas [30]. Research on agricultural soil has primarily focused on major grain-producing regions, high-yield agricultural areas, and renowned grain production bases [31–33], with little attention paid to regions specializing in unique agricultural products. Cangxi County in Sichuan Province is the main production area and original birthplace of kiwifruit in China, with approximately 39.5 thousand hectares of kiwifruit orchards and an annual yield of about 12.6 thousand tons. Its kiwifruit products are exported to countries such as Japan, Singapore, and France, thus establishing the kiwifruit industry as a key pillar of Cangxi County’s economic development [34]. The study focuses on the designated key planting area for red heart kiwifruit in Cangxi County. Significantly, soil contamination can impact the quality of kiwifruit, thereby affecting human health and impeding the high-quality development of the kiwifruit industry. A review of the relevant literature reveals that current research on soil heavy metals in this region is limited to environmental quality assessments [35], lacking analysis of the spatial distribution and sources of these metals. Understanding the spatial distribution and sources of soil heavy metals is crucial for regional agricultural production. Therefore, this study aims to examine the key kiwifruit planting area in Cangxi County, analyzing the spatial variation and distribution patterns of soil heavy metals. It employs the PMF and PCA/APCS models to identify and quantify the sources of soil heavy metal pollution, aiming to offer scientific insights for the safe cultivation of kiwifruit and the sustainable growth of the kiwifruit industry in the area.

## 2. Study area and methodology

### 2.1. Study area

**Fig 1:**
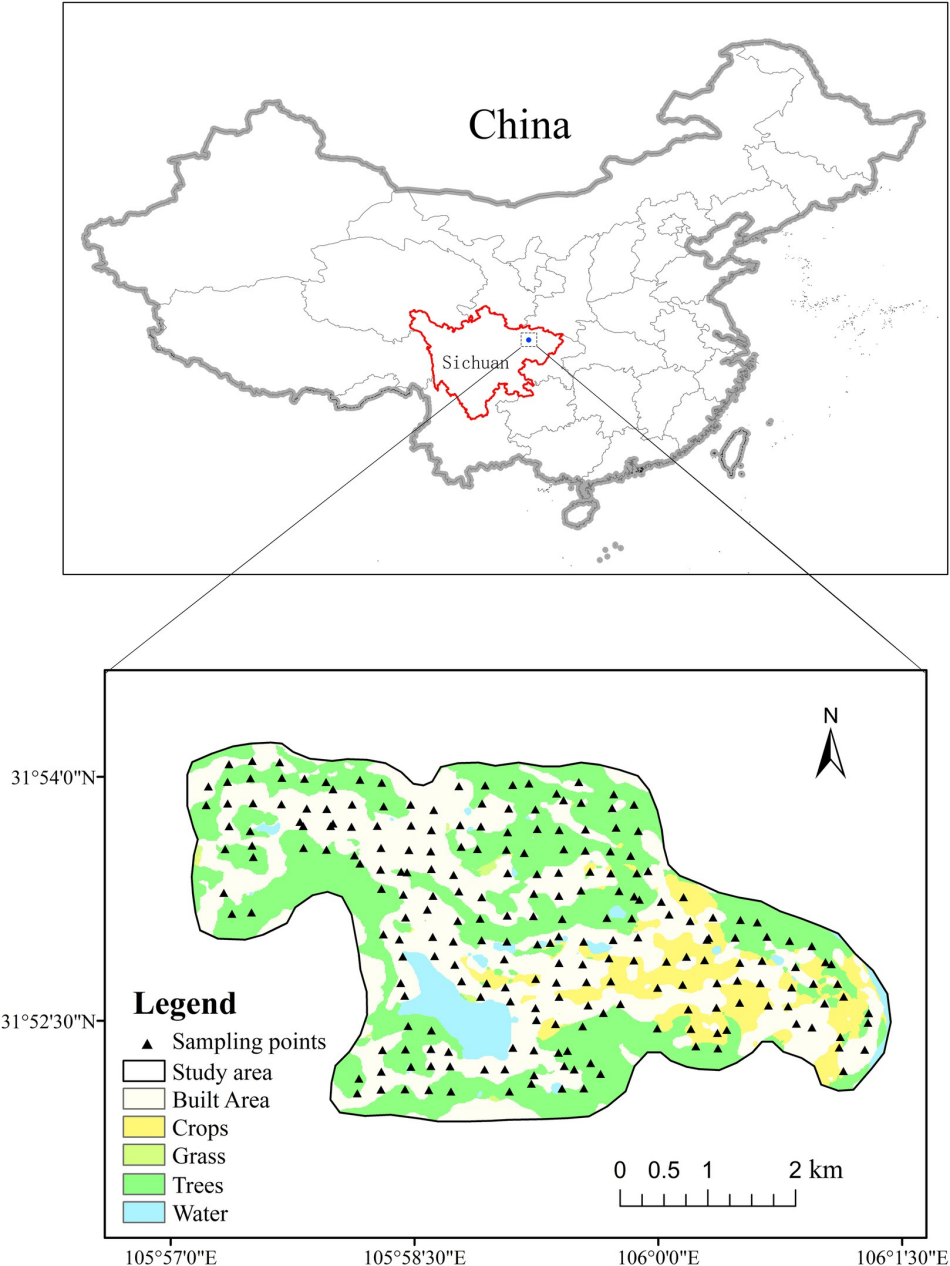
Study area location and sampling sites.

The study area, covering 17.53 km^2^ (Fig 1), is situated in the northeastern part of Sichuan Province, spanning latitudes 31°51′–32°55′ N and longitudes 105°57′–106°02′ E. With a terrain sloping from northwest to southeast, the area features gentle slopes, along with gullies and valleys more developed in the surroundings, gradually transitioning to broad and flat terrains at the center. It belongs to the mid-subtropical humid monsoon climate zone, with a mild climate and four distinct seasons. The average annual temperature is 16.9°C and the average annual rainfall is 1100mm [36].The eastern part is adjacent to the Yangjia River, which belongs to the water system of the Jialing River. The soil is dominated by Cambisol, which accounts for about 70% of the total area. The region’s geology primarily comprises sandstone, with Cretaceous mudstone as the parent material. The soil texture is predominantly loam, followed by light clay, purple sands, and sandy loam. Granular loam soil is prevalent, whereas blocky loam soil appears in lesser quantities. Soil organic matter content is generally low, influenced by the terrain’s slope and the depth of the tillage layer. The primary crops are rice, wheat, corn, and rapeseed, alongside the cultivation of economic crops such as vegetables, snow pears, walnuts, and kiwifruit. The land cover encompasses built area, crops, trees, water and grass.

### 2.2. Sample collection, preparation, and analysis

Utilizing a 1:10000 scale topographic map with pre-established sampling points as a fieldwork guide, navigate to the sampling points using Global Positioning System (GPS) technology and record their geographic coordinates. The selection of sampling sites deliberately avoided ridges, ditches, roadsides, and areas with unique terrain features, instead focusing on sampling in large agricultural fields, vegetable plots, forested regions, and riverbank slopes. Soil columns (0 ~ 20cm) of 3 ~ 5 sub-sampling points (radial) were collected within a radius of 50 m around the sampling point. After the soil of the sampling points was evenly mixed, 1.5 kg samples were taken according to the quartering method and loaded into a special sample bag. The samples were isolated with polyester bags. A total of 228 topsoil (cultivated layer) samples were collected at an average density of 16 pieces / km^2^ (Fig 1). The collected samples were sent to Chengdu Comprehensive Rock and Mineral Testing Center for analysis and determination.

The collected soil samples were air-dried, after which impurities such as gravel, and animal and plant residues were removed. The soil was then crushed using a wooden stick until it could pass through a sieve with a 2mm aperture. Subsequently, the sifted soil samples were finely ground using an agate mortar until they could pass through a sieve with a 0.149mm aperture (100 mesh) and were then placed in sealed bags for subsequent analysis. The sample analysis methods and quality control procedures were implemented in strict accordance with the “Specification of Testing Quality Management for Geological Laboratories” (DZ/T 0130–2006) and the “Specification of Multi-purpose Regional Geochemical Survey” (DD2005-01). Soil pH was measured using an ion meter, with the pH range of the measured samples ranging from 5.26 to 8.56, and an average value of 7.26, indicating neutral soil. Heavy metal testing was conducted through microwave digestion. An appropriate amount of soil sample was mixed with an HCl-HNO3 acid mixture, digested in a microwave system, and then treated with 2% potassium borohydride for mercury content analysis using an atomic fluorescence spectrometer (AFS-8220, Beijing Jitian, China) [37]. The contents of Cr, Cu, Pb, Ni, and Zn were analyzed using X-ray fluorescence spectrometry (ARL Perform, Thermo, Europe), a rapid analytical technique renowned for its waste reduction, low start-up, and operating costs [38]. To ensure the quality and accuracy of experimental results, blank and parallel samples were prepared throughout the experiment. National class a standard matters (GWB) were employed for quality control during the testing process. The recovery rates for the six elements ranged from 85% to 105%. The relative standard deviations (RSD) for all processed batches were below 5%. The detection limits for Cr, Cu, Pb, Ni, Zn, and Hg were 2, 1, 2, 2, 4, and 0.003 mg/kg, respectively.

### 2.3. Methodology of the study

#### 2.3.1. Pollution assessment

Quantitative assessment of pollution for a single heavy metal in a specific area is typically conducted using the single pollution index method, which reflects the multiple of exceeding the standard and the degree of pollution for the heavy metal, thereby identifying the primary pollutant in the area[39]. Its calculation formula is as follows [40]:

$$P_{i}=C_{i}/S_{i}$$ (1)

where P_i_ is the pollution index value of heavy metal i, C_i_ is the measured value of heavy metal i, S_i_ is the main standard value for environmental assessment of heavy metal i, and here is the soil background value of Sichuan Province [41].

The comprehensive pollution index method can reflect the overall pollution level of multiple heavy metal elements in the soil within a specific area.The calculation formula is as follows [40]:

$$P_{N}=\sqrt{[{\left(P_{max}\right)}^{2}+{\left(P_{ave}\right)}^{2}]/2}$$ (2)

where P_N_ is the heavy metal comprehensive pollution index value, P_max_ is the maximum value in P_i_, and P_ave_ is the mean of P_i_. The classification standard of heavy metal pollution is shown in Table 1 [42,43].

**Table 1:** Classification standard of soil heavy metal pollution.

Single pollution index	Pollution level	Comprehensive pollution index	Pollution level
P_i_≤1	no pollution	P_N_<0.7	no pollution
1<P_i_≤2	low pollution	0.7≤P_N_<1	warning line
2<P_i_≤3	medium pollution	1≤P_N_<2	light pollution
P_i_>3	high pollution	2≤P_N_<3	medium pollution
		P_N_≥3	heavy pollution

#### 2.3.2. Semivariogram and Kriging interpolation

A semivariogram is a unique function in geostatistical analysis primarily used to describe the spatial variation characteristics and intensity of regionalized variables. It reflects the changes in soil properties between observed values at different distances within a region and serves as an effective tool for analyzing the spatial variation and spatial structure of soil heavy metals[44]. The calculation formula is as follows [45]:

$$\gamma (h)=\frac{1}{2N(h)}\sum_{i=1}^{N(h)}{\left[Z(x_{i})-Z(x_{i}+h)\right]}^{2}$$ (3)

where γ(h) is a semivariogram value, h is the space separation distance of two sample points, also known as step, N(h) is the number of pairs of sample points with spacing h, x_i_ is the i-th point corresponding to the distance h, Z(x_i_) is the measured value at the x_i_ position, Z(x_i_+h) is the measured value at x_i_+h. The semivariogram curve includes four important parameters: nugget value, sill value, partial sill value and range. The nugget value represents the randomness of the regionalized variables. The sill value and the range are used to measure the magnitude of the regionalized variable change and the size of the autocorrelation range, respectively. The partial abutment value is the difference between the sill value and the nugget value.

Kriging interpolation is a method based on variogram theory and structural analysis that is used to perform a linear, unbiased, optimal estimation of regionalization variables in a given finite region. The semivariogram and its model fitting have a great influence on the accuracy of Kriging interpolation results. Selecting reasonable model parameters for interpolation can improve the accuracy of the results.

#### 2.3.3. PCA/APCS analysis

Principal component analysis (PCA) employs dimensionality reduction to transform a correlated set of original variables into new, linearly independent variables via orthogonal transformation. These new variables, aiming to maximize the explanation of the variances among the original variables, are identified as the principal components. Before principal component analysis, Kaiser-Meyer-Olkin (KMO) and Bartlett tests were used to evaluate whether the data was suitable for this method. The KMO test evaluates the degree of correlation among variables, yielding values ranging between 0 and 1. Values above 0.6 are generally deemed acceptable [46]. The Bartlett test is another measure of the correlation strength between variables based on the chi-square distribution. There is no correlation between the null hypothesis variables. If the P value is less than the significance level (P<0.05), we reject the null hypothesis, suggesting that the data is suitable for principal component analysis [47].

The principal component analysis (PCA) method can only qualitatively infer the number of possible pollution sources but cannot be directly used for source analysis [48], so it needs to be combined with absolute factor analysis-multiple linear regression receptor modelling (APCS-MLR) to perform a quantitative analysis. PCA/APCS receptor modelling has become a commonly used method in the field of pollution source identification, and it can derive the different pollution sources’ contribution. The calculation steps are as follows [49]:

$$Z_{ij}=(\frac{C_{ij-}\bar{C}}{\sigma_{j}})$$ (4)

$${\left(Z_{0}\right)}_{j}=\frac{0-\bar{C}}{\sigma_{j}}=-\frac{\bar{C}}{\sigma_{j}}$$ (5)

$${\left(A_{0}\right)}_{f}=\sum_{j=1}^{J}S_{fj}∙{\left(Z_{0}\right)}_{j}$$ (6)

$${APCS}_{f}={\left(AZ\right)}_{if}-{\left(A0\right)}_{f}\;f=1,\;2,\ldots \ldots ,\;F;$$ (7)

$$C_{j}={\left(r_{0}\right)}_{j}+\sum_{k=1}^{F}r_{kj}*{APCS}_{k}$$ (8)

where Z_ij_ is the standardised concentration value, C_ij_ is the measured value of heavy metal concentration, $\bar{C}$ and σ_j_ are the mean concentration and standard deviation of heavy metal elements, respectively, (Z_0_)_j_ is the standardised value when the concentration of heavy metal elements is 0, (A_0_)_f_ is the f principal component score value when the concentration is 0, S_fj_ is the coefficient of the factor scores of the f principal components of j heavy metals, where j is the heavy metal serial number, (r_0_)_j_ is the constant term of the multiple regression, r_kj_ is the regression coefficient of pollution source k on heavy metal j, APCS_k_ is the absolute principal factor score, and r_kj_*APCS_k_ is the contribution of source k to C_j_.

The contribution of each source of soil heavy metals was calculated as follows [50]:

$${PC}_{kj}=\frac{|r_{ij}*\bar{{APCS}_{k}}|\times 100\%}{|{\left(r_{0}\right)}_{j}|+\sum_{k=1}^{F}\left|r_{ij}*\bar{{APCS}_{k}}\right|}$$ (9)

where (r_0_)_j_ is the unidentified source contribution (unknown source).

The contribution of unidentified sources can be calculated as follows [50]:

$${PC}_{kj}=\frac{|{\left(r_{0}\right)}_{j}|\times 100\%}{|{\left(r_{0}\right)}_{j}|+\sum_{k=1}^{F}\left|r_{ij}*\bar{{APCS}_{k}}\right|}$$ (10)

where PC_kj_ is the contribution of source k to heavy metal j, and $\bar{{APCS}_{k}}$ is the mean value of APCS of all samples.

#### 2.3.4. PMF model

The model decomposes the original matrix (X_ij_) into a matrix of source contribution factors (G_ik_) and a matrix of source compositional spectral factors (F_kj_) as well as a residual matrix (E_ij_), and quantitatively identifies the contribution of each source through the results of the solution [51], with the following equation [52]:

$$X_{ij}=\sum_{k=1}^{P}G_{ik}F_{kj}+E_{ij}$$ (11)

where X_ij_ is the content of the jth heavy metal element in the ith sample, G_ik_ is the contribution of the kth source to the ith sample, F_kj_ is the content of the jth heavy metal element in the kth source, and E_ij_ is the residual of the jth element in the ith sample.

The optimal matrices G and F are obtained by decomposing the original matrix X of the PMF model to minimise the objective function Q. The objective function Q is calculated as follows [52]:

$$Q=\sum_{i=1}^{n}\sum_{j=1}^{m}{\left(\frac{E_{ij}}{U_{ij}}\right)}^{2}$$ (12)

where U_ij_ is the magnitude of uncertainty in the content of the jth element in the ith sample. The calculation formula is as follows [53]:

$$U_{ij}=\left\{ \begin{array}{r} 5/6\times MDL,\;\partial \leq MDL\\ \sqrt{{\left(\delta \times \partial \right)}^{2}+{MDL}^{2}},\;\partial >MDL \end{array}\right.$$ (13)

where δ is the relative standard deviation, ∂ is the concentration of heavy metal elements, and MDL is the method detection limit.

### 2.4. Statistical analysis

The descriptive statistics (maximum, minimum, mean, standard deviation, coefficient of variation, etc.) of heavy metals and the evaluation of the pollution index were done in Excel 2016. The semivariogram analysis and the establishment of theoretical model were completed by GS + 9.0. The spatial distribution map of soil heavy metals based on Kriging interpolation method was made by ArcGIS 10.5. Pearson correlation analysis was done in the software Origin 2022, and source resolution was done in the software SPSS 23.0 (PCA/APCS model) and EPA PMF 5.0 (PMF model), respectively.

## 3. Results and discussion

### 3.1. Soil heavy metal concentrations

**Fig 2:**
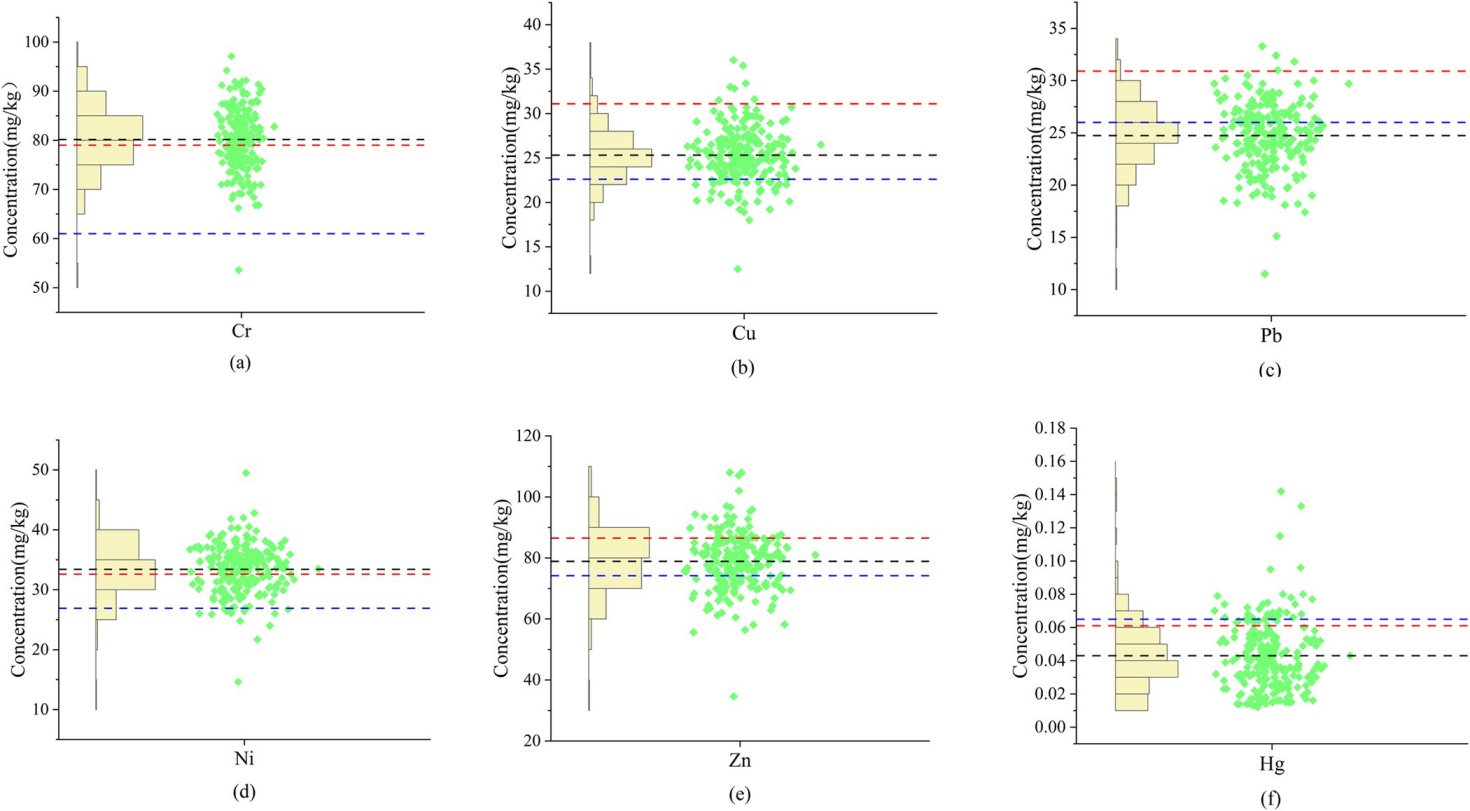
Columnar scatter diagram of heavy metal concentration in soil. (**a**) Cr; (**b**) Cu; (**c**) Pb; (**d**) Ni; (**e**) Zn; (**f**) Hg. The red line represents the background value of soil in Sichuan Province; the blue line represents the background value of soil in China; the black line represents the average concentration of heavy metals.

The statistical properties for heavy metals present in the surface soil of the study area are presented in Table 2. The average concentrations of Cr, Cu, Pb, Ni, Zn, and Hg are 80.156, 25.313, 24.736, 33.404, 78.887, and 0.043 mg/kg, respectively. None of these six elements exceed the threshold values set by the “Soil Environmental Quality Agricultural Soil Pollution Risk Control Standard” (GB 15618–2018). A column scatter plot showing soil heavy metal concentrations was displayed in Fig 2. Based on the average concentrations, Cr and Ni exceed Sichuan Province’s soil background values, while Hg and Pb are lower than China’s soil background values. For each measurement point, the concentrations of Cr, Cu, Pb, Ni, Zn, and Hg had 141, 7, 4, 140, 40, and 39 instances, respectively, exceeding the background values for Sichuan Province, and 227, 190, 76, 214, 165, and 40 instances, respectively, exceeding the background values for Chinese soil. The coefficient of variation serves as a measure of the average degree of variation across individual sample points [40]. A higher coefficient of variation indicates greater dispersion within the data distribution, meaning there is a more significant variance in the spatial distribution of heavy metal concentrations. Under the classification criteria, a coefficient of variation below 20% signifies low variability, between 20% and 50% indicates moderate variability, from 50% to 100% suggests high variability, and above 100% denotes extreme variability [54]. Cr, Cu, Pb, Ni, and Zn all fall into the low variability category, indicating that the spatial distribution differences of these five heavy metals are relatively small. Hg falls into the moderate variability category, suggesting that certain specific sampling points exhibit higher concentrations, possibly indicating localized point source pollution [55].

**Table 2:** Descriptive statistics of soil heavy metals in the study area.

Heavy metal	Cr	Cu	Pb	Ni	Zn	Hg
Max/mg·kg^-1^	97.100	36.000	33.300	49.500	108.000	0.142
Min/mg·kg^-1^	53.600	12.500	11.500	14.600	34.600	0.012
Mean/mg·kg^-1^	80.156	25.313	24.736	33.404	78.887	0.043
Standard deviation	6.073	3.039	3.107	3.995	9.268	0.020
Coefficient of variation /%	7.576	12.007	12.561	11.959	11.748	46.648
^a^ Soil background values for Sichuan Province/mg·kg^-1^	79	31.1	30.9	32.6	86.5	0.061
^a^ Soil background values for China/mg·kg^-1^	61	22.6	26	26.9	74.2	0.065
^b^ Risk screening value/ mg·kg^-1^	300	200	140	100	250	0.6

^a^ Central Station of Environment Monitoring of China, Background values of soil elements in China, 1990 [41].

^b^ Ministry of Ecology and Environment of the People’s Republic of China, Soil environmental quality risk control standard for soil contamination of agriculture land, 2018 [56].

### 3.2. Spatial variability and distribution pattern of soil heavy metals

Semivariogram analysis necessitates that the sample data either strictly follow a normal distribution or closely approximate one. Failing to meet this condition can lead to the scale effect, which distorts the actual variogram values and diminishes the accuracy of estimations [57]. The test for soil heavy metal distribution characteristics is presented in Table 3, and the Kolmogorov-Smirnov (K-S) test was performed using SPSS. The test showed that Cr, Ni, and Zn follow a normal distribution with p > 0.05, while Cu, Pb, and Hg do not. Beyond the K-S test, the skewness and kurtosis coefficients can also be used to determine the adherence of sample data to a normal distribution. The skewness coefficient measures the asymmetry of the heavy metal concentration distribution, while the kurtosis coefficient measures the concentration around the mean value. The closer these coefficients are to 0, the closer the data is to a normal distribution. The skewness coefficients of Cu and Pb fall within the interval [–1,1], indicating an approximate adherence to a normal distribution [58]. After logarithmic transformation, the skewness coefficient and kurtosis coefficient of Hg are -0.342 and -0.166, respectively, indicating adherence to a normal distribution.

**Table 3:** Testing of distribution characteristics of soil heavy metals.

Element	Cr	Cu	Pb	Ni	Zn	Hg
Skewness	-0.337	0.131	-0.476	-0.362	-0.28	-0.342^a^
Kurtosis	1.016	1.844	1.212	2.461	2.507	-0.166^a^
K-S test	0.047	0.065	0.067	0.056	0.052	0.065
K-S *p*	0.200	0.020	0.015	0.078	0.200	0.022
Distribution	normal	normal	normal	normal	normal	lognormal

^a^ Skewness and kurtosis for Hg are normalised values.

According to the principle of maximum coefficient of determination (r^2^) and minimum residuals (RSS) [59], the theoretical model and its parameters are shown in Table 4. The theoretical models for the elements are exponential, indicating characteristics of clustered distribution. The coefficients of determination, r^2^, are all greater than 0.7, among which those for Pb and Ni are greater than 0.9, indicating a good fit. The nugget value (Co) is the half-variance value at zero spacing (h = 0), which is mainly caused by the measurement error and the variation of random factors such as fertiliser application at less than sampling scale, crop, management level, etc. [60]. The size of the nugget values of the six elements is in the order of Zn (15)>Ni (7.61)>Cr (5.8)>Pb (5.31)>Cu (1.6)>Hg (0.0362), which indicates that there is some measurement error, but the variation caused is not large. The nugget coefficient is the ratio of the nugget value and the abutment value, indicating the degree of spatial correlation of the system variables. If the nugget coefficient (Co/Co+C) is higher than 75%, the spatial correlation of the variable is weak; a nugget coefficient (Co/Co+C) between 25% and 75% indicates moderate spatial correlation; and a nugget coefficient less than 25% indicates strong spatial correlation [61]. The nugget coefficients for Pb and Ni are 49.95% and 44.71%, respectively, showing a moderate spatial correlation, indicating that the spatial distribution of Pb and Ni may be influenced by both natural and anthropogenic factors [62]. The nugget coefficients for Zn, Cu, Hg, and Cr are all less than 25%, demonstrating a strong spatial correlation, suggesting that their spatial distribution is mainly influenced by natural factors. The variance range is the interval distance when the semi-variance function just reaches a relatively stable state, within which the variables have spatial correlation, and beyond which there is no spatial correlation. There are some differences in the variance ranges of the elements, with Pb having the largest variance range of 501 m and Cr having the smallest variance range of 131 m.

**Table 4:** The theoretical model parameters of soil heavy metal semivariogram.

Element	Theoretical model	Nugget (Co)	Sill (Co+C)	Nugget coefficient (%)	Range (m)	Residual (RSS)	r^2^
Cr	Exponential	5.8	38.71	14.98	131	9.230	0.765
Cu	Exponential	1.6	9.77	16.38	215	1.190	0.884
Pb	Exponential	5.31	10.63	49.95	501	0.833	0.944
Ni	Exponential	7.61	17.02	44.71	386	1.930	0.936
Zn	Exponential	15	90.83	16.51	152	82.900	0.778
Hg	Exponential	0.0362	0.2374	15.25	172	4.64E-04	0.807

**Fig 3:**
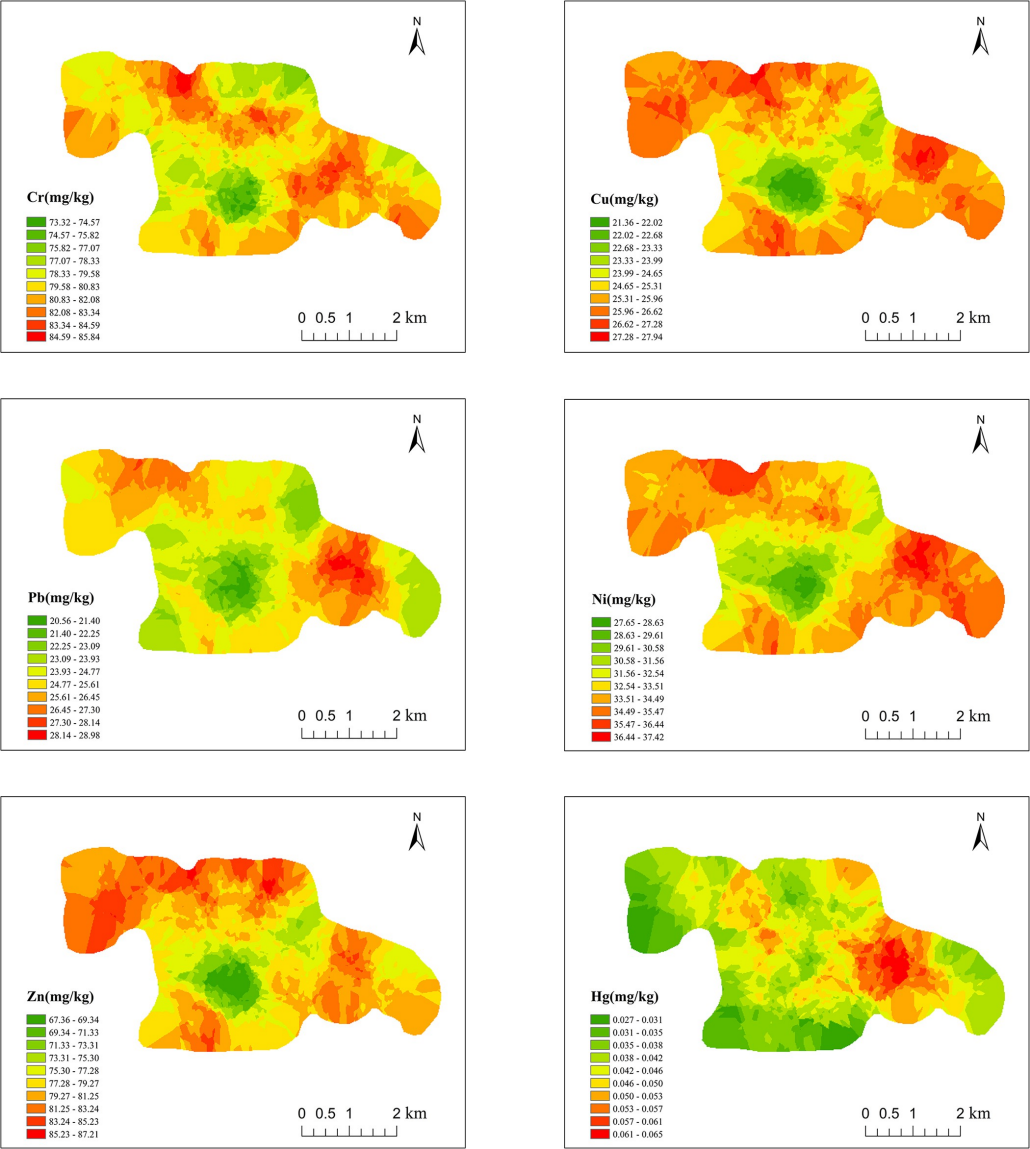
Spatial distribution of soil heavy metals.

The Kriging spatial interpolation of soil heavy metals was carried out by using the theoretical model parameters of semivariogram, and the results are shown in Fig 3. For each element, the high-value areas of Cr are mainly in the north and east, with fewer and scattered low-value areas. The high-value areas of Cu are mainly in the north and south, with the low-value areas concentrated in the center. The high-value areas of Zn are in the north, with the low-value areas distributed in the center. The high-value areas of Pb are mainly in the east, with the low-value areas primarily in the center. The high-value areas of Ni are more scattered, with most of them located in the east. The high-value areas of Hg are predominantly in the east, and the low-value areas are extensive, mainly in the east and south. In general, the spatial distribution of Cr, Cu, Zn, Pb and Ni is similar, with high values concentrated in the north and east and low values in the centre. The study area is dominated by agricultural development, the land use type is mostly agricultural land, the eastern region is mainly arable land, and the high concentration of heavy metals may be due to agricultural production activities. The northern part is mostly construction land, and the accumulation of heavy metals may be caused by the dumping of villagers’ domestic waste and motor vehicle driving. The low-value areas are reservoirs with little anthropogenic activity and generally low concentrations of heavy metals. The spatial distribution of Hg elements varies somewhat, with a wide range of low-value areas and high concentrations in only a few areas, which may be due to localised activities of villagers, such as the application of unqualified fertilisers and the exceeding of the Hg content in the water used for irrigation.

### 3.3. Assessment of heavy metal pollution of soil

**Fig 4:**
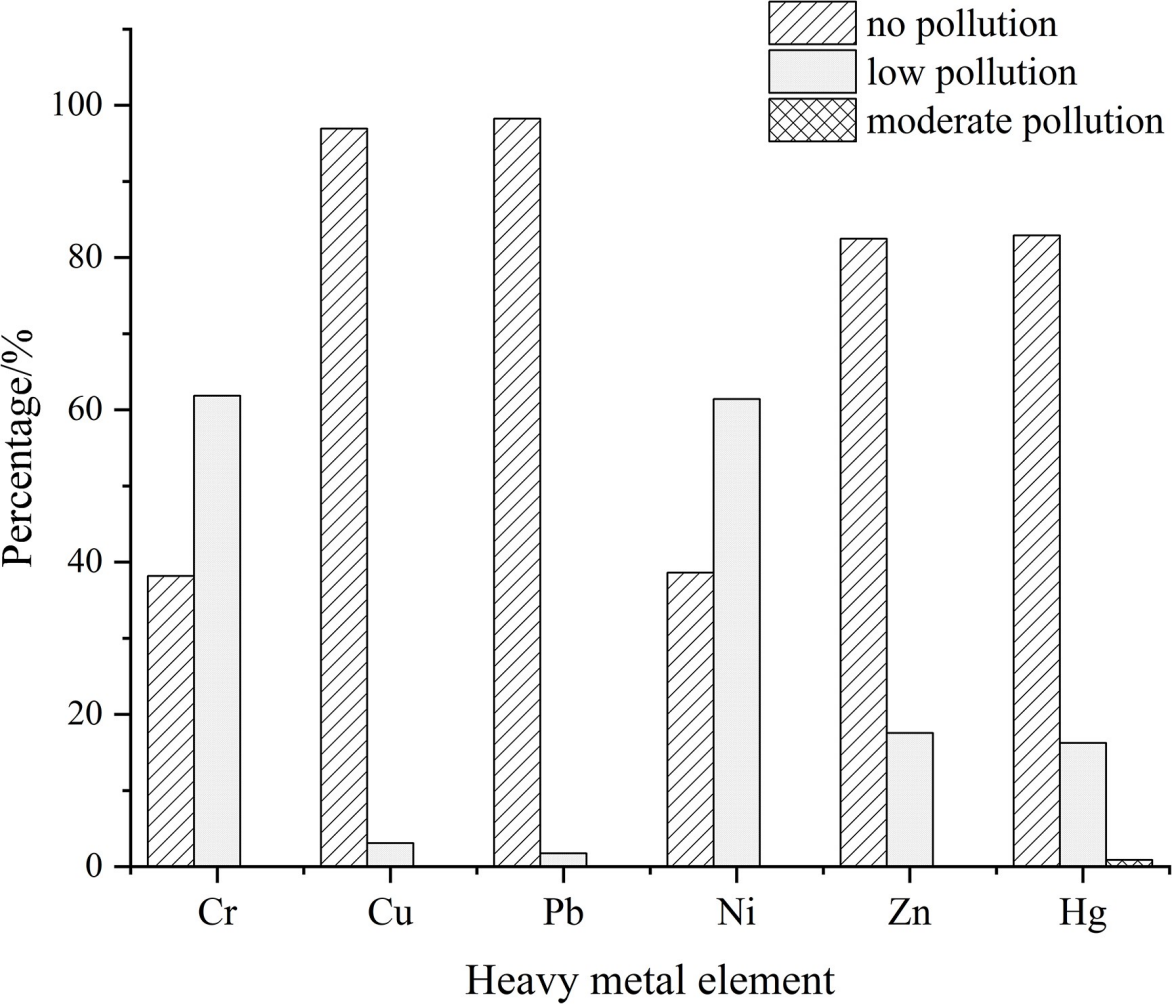
Classification of single pollution degree of heavy metal elements in soil at sampling points.

The results of the heavy metal pollution Assessment were shown in Table 5. The average value of the single pollution index of six heavy metal elements in the study area was Ni (1.025) > Cr (1.015) > Zn (0.912) > Cu (0.814) > Pb (0.801) > Hg (0.709). According to the grading standard of pollution degree, Cr and Ni were low pollution.Cu, Pb, Zn, and Hg were no pollution. Among all sampling points, about 97%, 98%, 82%, and 83% of the points of Cu, Pb, Zn, and Hg showed no pollution; about 62% and 61% of the points of Cr and Ni showed low pollution; Cr, Ni, Cu, Pb, and Zn did not reach medium pollution; and only two points of Hg showed medium pollution, which may be the reason for the large coefficient of variation of Hg (Fig 4).

**Table 5:** Evaluation results of heavy metal pollution.

Single pollution index							Comprehensive pollution index
Heavy metal	Cr	Cu	Pb	Ni	Zn	Hg	
Max	1.229	1.158	1.078	1.518	1.249	2.328	1.85 0.57 0.994
Min	0.678	0.402	0.372	0.448	0.400	0.197	
Mean	1.015	0.814	0.801	1.025	0.912	0.709	
Degree of contamination	Low pollution	No pollution	No pollution	Low pollution	No pollution	No pollution	Warning line

**Fig 5:**
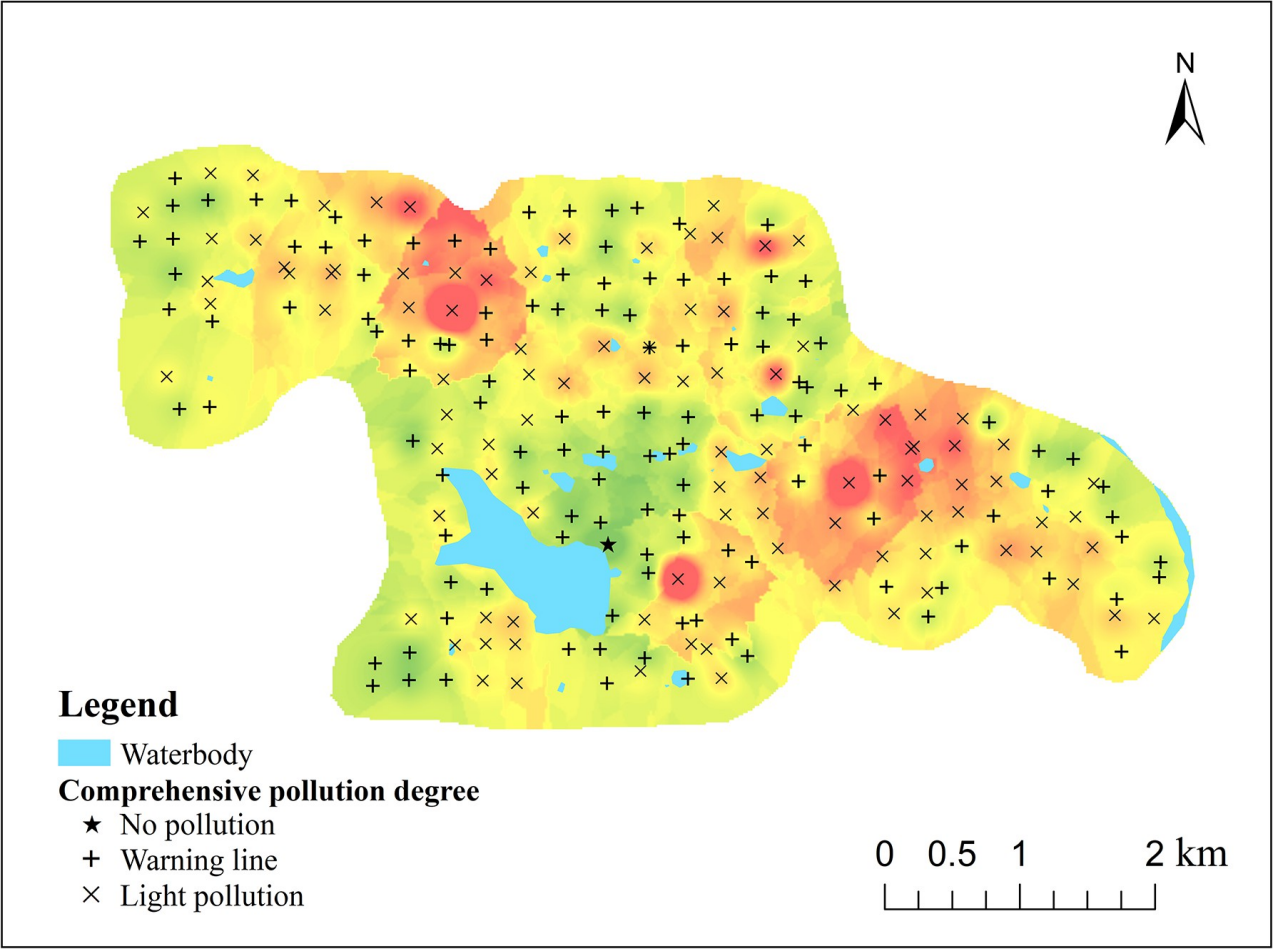
Comprehensive pollution degree of heavy metals in soil.

The mean value of the comprehensive pollution index was 0.994, which is in the warning line range according to the grading criteria. Among all sampling points, the comprehensive pollution index ranged from 0.57 to 1.85, with 55.7% and 43.9% of the points at the warning line and light pollution levels, respectively, and only one point was non pollution. The spatial distribution of integrated soil heavy metal pollution is shown in Fig 5, with the high-value areas mainly in the north and east and the low-value areas mainly around the reservoirs, which basically coincides with the high-value and low-value areas in the spatial distribution map of heavy metal concentrations (Fig 3). Most of the points within the high-value areas showed light pollution levels, indicating that these two areas may be subject to light anthropogenic pollution. The pollution level around the reservoir is low, and most of them are at the alert level. As mentioned in the previous content, the high-value areas are construction land and arable land respectively, indicating that human agricultural activities and transport may be the two major anthropogenic sources of soil heavy metal pollution in the study area, which will be analysed in the following.

### 3.4. Source analysis of heavy metals in soils

#### 3.4.1. Correlation analysis

**Fig 6:**
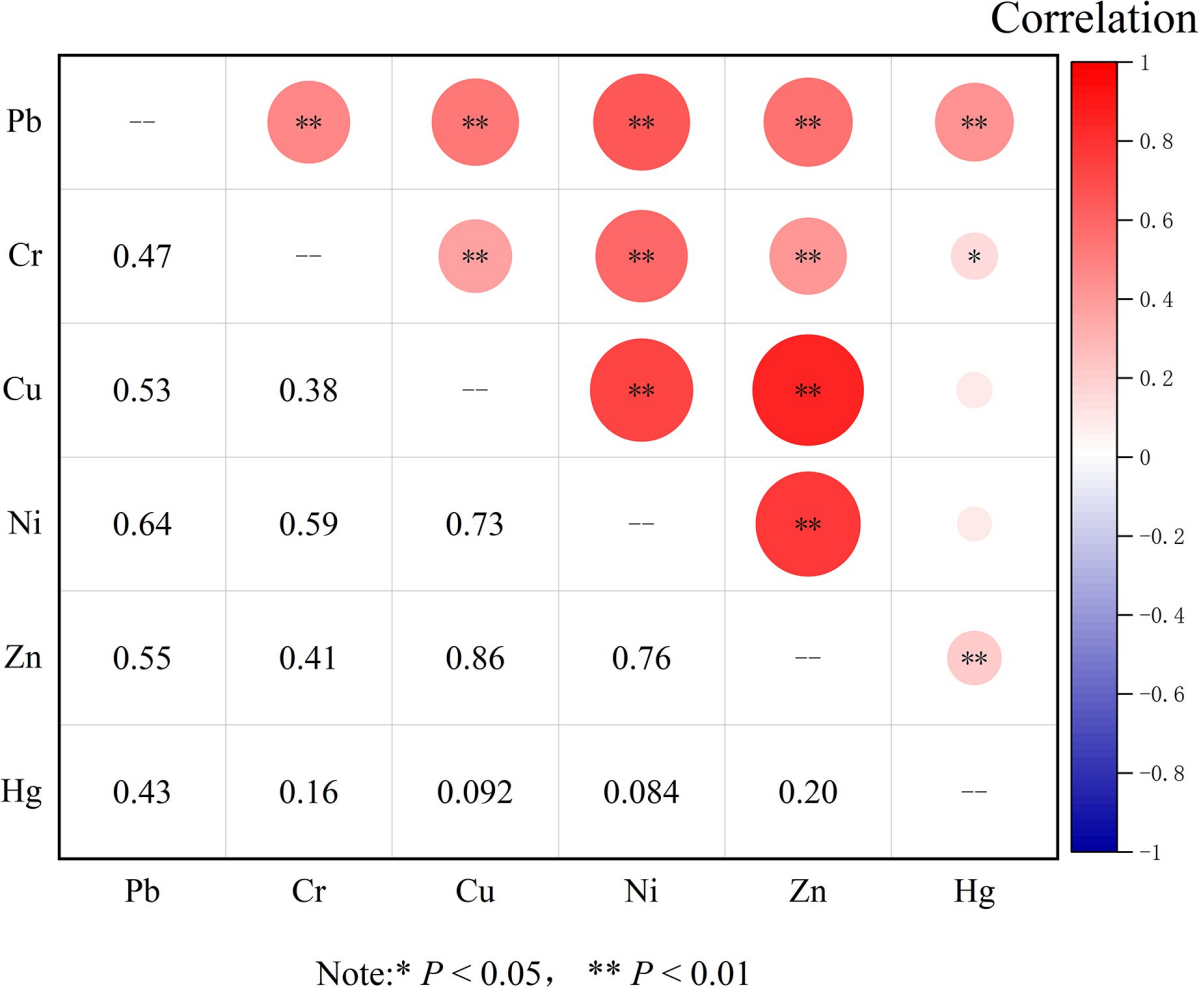
Soil heavy metal correlation coefficients.

The correlation analysis can effectively reflect the connections between various heavy metals, helping to infer their sources. The range of correlation coefficient (*r*) values is [–1, 1], where |*r*| between 0–0.1 indicates no correlation, 0.1–0.3 indicates weak correlation, 0.3–0.5 indicates moderate correlation, and 0.5–1 indicates strong correlation [46]. If there is a significant positive correlation between elements, it indicates that these elements have the same or similar pollution sources [63,64]. The analysis results were shown in Fig 6. There is a strong or moderate correlations between Pb, Cr, Cu, Ni, and Zn elements, and there is a highly significant positive correlations at the 0.01 confidence level, suggesting that these elements may have the same or similar pollution sources. Hg-Pb (*r* = 0.43), Hg-Zn (*r* = 0.20) show significant positive correlations at the 0.01 level, while Hg-Cr (*r* = 0.16) shows a significant positive correlation at the 0.05 level, although the correlations are not strong. The correlation coefficients for Hg-Cu (*r* = 0.092) and Hg-Ni (*r* = 0.084) are close to 0, indicating no correlation, suggesting that Hg may have different pollution sources compared to other elements.

#### 3.4.2. Identification of pollution sources with PCA/APCS

Kaiser-Meyer-Olkin (KMO) and Bartlett’s test were carried out after standardisation of the raw data and analysed by SPSS. The coefficient of the KMO test was 0.742>0.6, and the *p* was 0.000<0.05, which indicated that it was statistically significant and suitable for principal component analysis. According to the principle that the eigenvalue needs to be greater than 1, two principal components were extracted, with eigenvalues of 3.237 and 1.315, respectively, and the cumulative contribution rate is 75.868%, indicating that these two principal components represent most of the data (Table 6). The rotated factor loadings are as follows. Typically, factor loadings ranging from 0.3 to 0.5 are considered weak, 0.5 to 0.75 are moderate, and above 0.75 are strong loadings [65]. The contribution rate of PC1 is 53.957%, showing strong positive loadings for Cu (0.897), Ni (0.919), and Zn (0.891), while exhibiting moderate positive loadings for Cr (0.616) and Pb (0.644). PC2 contributes 21.911%, demonstrating a strong positive loading for Hg (0.954). The PCA analysis results suggest that Cr, Cu, Pb, Ni, and Zn may share a common source, whereas Hg originates from a different source, consistent with the findings of the correlation analysis.

**Table 6:** Component matrix after rotation for principal component analysis of soil heavy metals.

Heavy metal	PC1	PC2
Cr	**0.616** ^a^	0.241
Cu	**0.897**	0.000
Pb	**0.644**	0.571
Ni	**0.919**	0.080
Zn	**0.891**	0.115
Hg	0.017	**0.954**
Eigenvalue	3.237	1.315
Contribution/%	53.957	21.911
Cumulative contribution/%	53.957	75.868

^a^ Bold = loadings>0.50.

**Fig 7:**
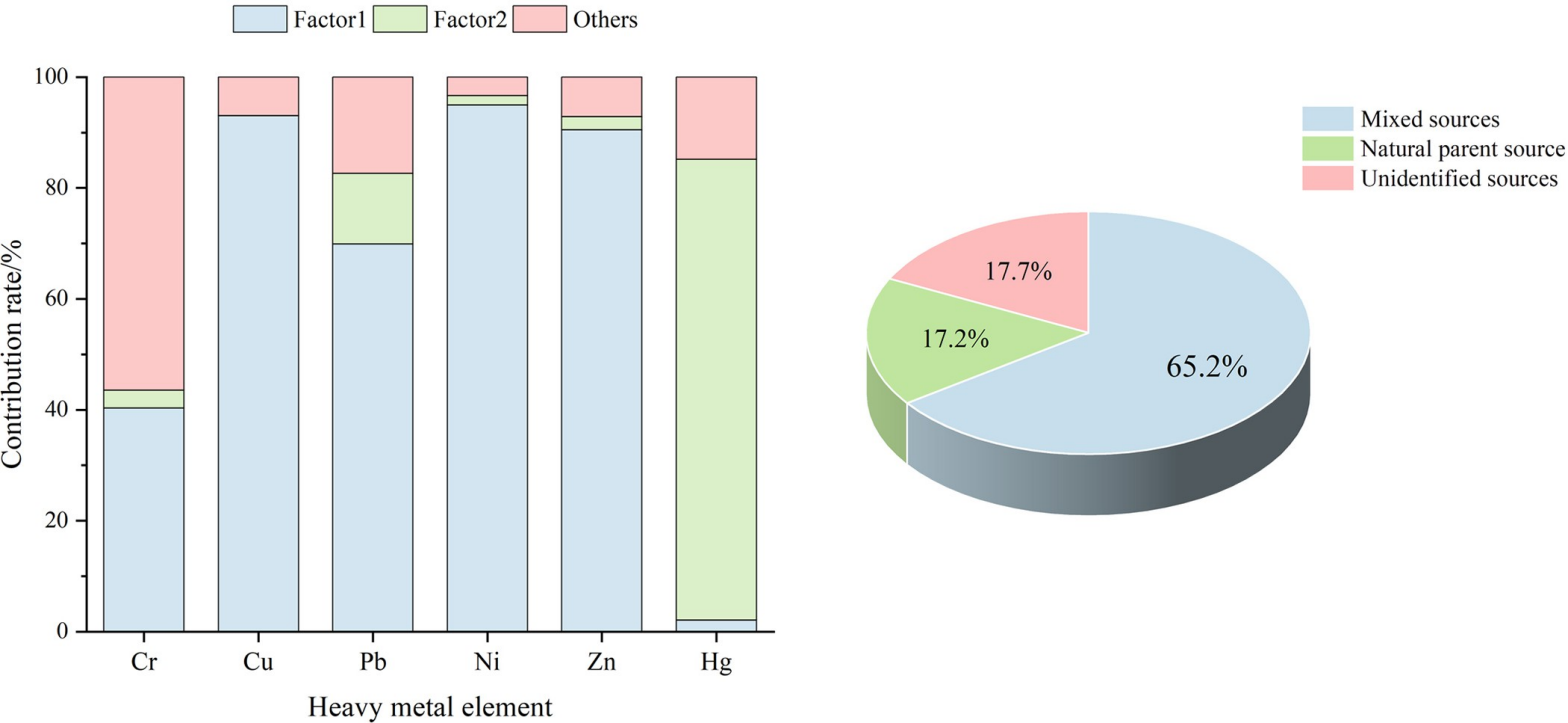
Contribution of PCA/APCS to the source analysis of soil heavy metal contamination.

According to the results of the heavy metal pollution Assessment (Table 5), Cr and Ni are mildly polluted, and Cu, Pb, and Zn are also polluted at certain points. Relevant studies have shown that Cr, Ni, Cu, Pb, and Zn mainly originate from irrigation and fertilisation, with high Pb and Cr content in commercial organic fertilisers and high Cu and Zn content in livestock manure [66,67]. In addition, automobile exhaust, worn tyres, and engine parts release Cu, Ni, and Pb, while Zn, as an important additive in the vulcanization process, accounts for 0.4–4.3% of tyre tread rubber, and tyre wear also contributes to the accumulation of Zn [68,69]. The study area is predominantly an agricultural area with convenient transportation conditions, is close to towns and cities, and is located near National Highway 212. Therefore, it is inferred that PC1 is a mixed source of agriculture and transportation. From the descriptive statistics of heavy metals (Table 2), the average content of elemental Hg was lower than the soil background values in Sichuan Province and China. Combined with the single-factor pollution evaluation, Hg was overall non-polluted and in a clean state, and some studies have shown that the concentration of soil Hg was lower than the local soil background value, and there may be natural sources [20]. From this, PC2 was judged to be a natural parent source. The combined contribution rate of soil heavy metal pollution sources based on the PCA/APCS model is shown in Fig 7, with a contribution rate of 65.2% from mixed agricultural and transportation sources; 17.2% from natural parent sources; and 17.7% from unidentified sources. The low contribution rate of unidentified sources indicates only minor impacts, which may be mixed pollution from dumping of domestic waste, sewage irrigation, and atmospheric deposition [70]. The highest contribution rate of mixed sources from agriculture and transportation indicates that the input of heavy metals from agricultural production and transportation should be emphasised. In agricultural production, it is imperative to enhance the monitoring of fertilizers and pesticides, increase the application of organic and biological fertilizers, opt for pesticides that are highly efficient, low-toxicity, and low-residue, and regulate heavy metal content. In the realm of transportation, priority should be given to unleaded gasoline, the promotion of gas fuels, and the utilization of lubricants or fuel additives that comply with established standards.

#### 3.4.3. Pollution source apportionment using PMF model

The heavy metal concentrations and uncertainty data were imported into EPA PMF 5.0, and the signal-to-noise ratios (S/N) of each element were all greater than 1, which was categorised as "strong". According to the production and living conditions in the study area, 2–4 factors were set to carry out the calculation for several iterations, and the R^2^ was greater than 0.7 when the number of factors was 3, which showed a good fitting effect. The calculated residual values are all within the range of +3 and -3, indicating good modelling.

**Fig 8:**
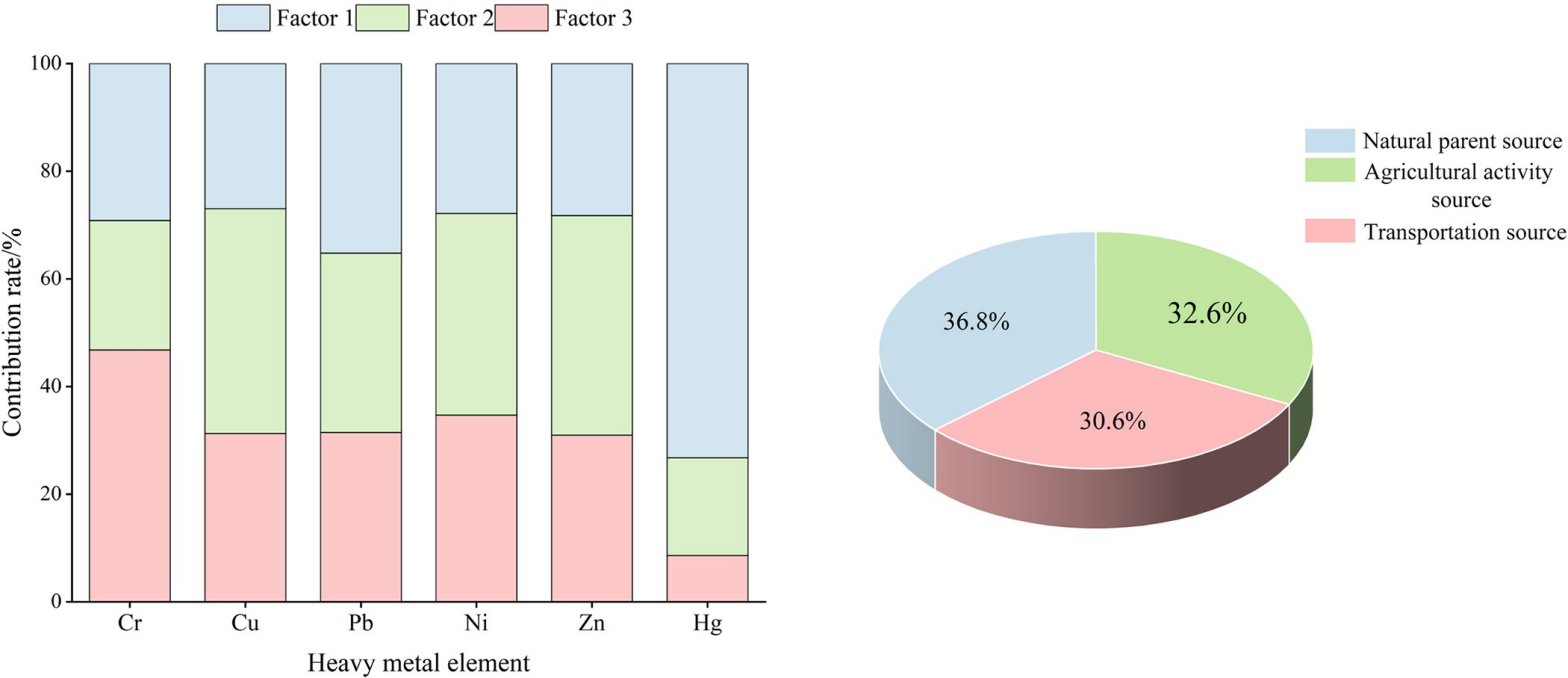
Contribution of soil heavy metal sources in PMFmodel.

The analysed results were shown in Fig 8. Factor 1 has the highest contribution to Hg, which is 73%. From previous studies, the sources of Hg are more complex; coal combustion, non-ferrous metal mining, animal and poultry manure, and fertiliser application may lead to the enrichment of Hg [71,72], and about 45% of Hg emissions in China come from non-ferrous metal smelting, 38% from coal combustion, and 17% from other activities [73]. There are no large-scale factories or mines in the study area, and the overall concentration of elemental Hg is low, suggesting that it is less affected by the above sources, and factor 1 is considered to be a natural parent source. Factor 2 contributed more to Cu, Pb, Ni, and Zn with 42%, 33%, 37%, and 41%, respectively. Based on the previous discussion, all four elements are associated with agricultural activities. The use of herbicides or pesticides can lead to the accumulation of Cu, as Cu is a common component in Bordeaux mixtures (fungicides), which are widely used in the control of pests and diseases in orchards and gardens [68,74]. Feed additives contain Pb and Zn, which will enter the soil through animal manure. In addition, phosphorus fertilisers contain some Pb and Zn, and irrational fertilisation will lead to the accumulation of Pb and Zn [75,76]. The study area is the key planting area of red kiwifruit planned by Cangxi County. Fruit trees are prone to pests and diseases, and the excessive use of pesticides by the farmers to ensure the yield may have led to the accumulation of Cu. After the field survey, the farmers in this area are more traditional in their operations and use organic fertilisers for fertilisation, which may have led to enrichment of Zn and Pb. In agricultural soils, Ni is generally considered to originate from natural sources [43,49,51]. However, studies have indicated that the natural geological environment can be affected by agricultural production, which in turn promotes the transfer of Ni from the geological background into the soil [69,77]. The spatial distribution analysis of Ni reveals that areas with high concentrations are predominantly covered by crops, suggesting that Ni accumulation may be significantly influenced by agricultural activities. Therefore, it is inferred that factor 2 is the source of agricultural activities. Factor 3 had the highest contribution of 47% to Cr. Related studies have shown that Cr accumulation is related to traffic road density, mainly originating from tyre wear [78,79]. The study area is adjacent to National Highway 212, which has a high traffic flow, and since the transportation of fruit products has certain requirements on roads and the transportation conditions in the area are more convenient, it is inferred that factor 3 is a source of transportation.

#### 3.4.4. Comparison of PCA /APCS and PMF models

A comparison of the fitting effects (R^2^) of the predicted and measured concentrations of each heavy metal in the PCA/APCS and PMF models is shown in Table 7. The R^2^ of Cr, Cu, Pb and Zn in the PMF model were all larger than that in the PCA/APCS model, and the mean value of R^2^ in the PMF model was 0.862 higher than that in the PCA/APCS model with a mean value of R^2^ of 0.757, and the lowest value of R^2^ in the PMF model was 0.742 (Pb), and the lowest value of R^2^ in the PCA/APCS model was 0.433 (Cr). The lowest value of R^2^ in the PMF model is 0.742 (Pb), and the lowest value of R^2^ in the PCA/APCS model is 0.433 (Cr). By comparison, overall, the PMF model is superior to the PCA/APCS model in terms of fitting effectiveness.

**Table 7:** Comparison of R^2^ and source analysis contribution rate (%) between PCA/APCS and PMF model.

Heavy metal	PCA/APCS				PMF			
	R^2^	Mixed sources	Natural parent source	Unidentified sources	R^2^	Agricultural activity source	Transportation source	Natural parent source
Cr	0.433	40.34	3.24	56.42	0.993	24.06	46.79	29.15
Cu	0.802	93.06	0.01	6.93	0.887	41.75	31.28	26.97
Pb	0.739	69.94	12.71	17.36	0.742	33.31	31.48	35.21
Ni	0.849	94.98	1.70	3.32	0.829	37.47	34.70	27.84
Zn	0.806	90.50	2.39	7.11	0.872	40.81	30.96	28.23
Hg	0.910	2.13	83.07	14.79	0.849	18.15	8.61	73.24
Combined contribution rate	—	65.16	17.19	17.65	—	32.59	30.64	36.77

The PCA/APCS and PMF models exhibit certain similarities in source apportionment results. The PCA/APCS model extracted two principal components with eigenvalues greater than 1, revealing mixed sources of agriculture and traffic, along with a natural parent source. In a similar vein, the PMF model identified agricultural activities, transportation emissions, and a natural parent source as pollution sources, suggesting that the heavy metal sources in the study area are linked to agriculture, transportation, and natural background. Concurrently, the PCA/APCS model associated elements such as Cr, Cu, Pb, Ni, and Zn with mixed sources, while Hg was linked to the natural parent source, which is in line with the findings from the PMF model.

The combined contribution rate of each source in the two models is somewhat different. The combined contribution rate of each source in the PCA/APCS model is mixed source (65.16%)>natural parent source (17.19%)>unidentified sources (17.65%), and that of the PMF model is natural parent source (36.77%)>agricultural activity source (32.59%)>transportation source (30.64%). The joint contribution of agricultural activity sources and transportation sources in the PMF model (63.23%) is close to that of the mixed sources in the PCA/APCS model, but the contribution of natural parent material sources is greater than that of the PCA/APCS model. The difference in the combined contribution of the sources may be caused by the different factor decomposition processes of the two models. The PCA/APCS model is based on the principle of principal component analysis, which usually selects factors with eigenvalues greater than 1 and cumulative variance contributions greater than 75% as principal components, whereas the PMF model takes into account uncertainty and weighting, and the decomposition process has a non-negative constraint[80]. In addition, the contribution rates of pollution sources to each heavy metal element in the two models are different; the contribution rates of natural parent sources to Cu, Ni, and Zn in the PCA/APCS model are all lower than 3%, and even to Cu, they are only 0.01%, while the contribution rates of the mixed sources to the three heavy metals are more than 90%; the contribution rates of each source to Cu, Ni, and Zn in the PMF model are more uniform, and there are no excessive high and low values. excessive high and low values, which may be related to the difference in data input between the two models. The PCA/APCS model considers only the pollutant concentration in the data input, whereas the PMF model takes into account the uncertainty associated with the concentration and the method detection limit at each point in addition to the pollutant concentration, and this point-by-point error estimation provides robust results [52].

Both the PCA/APCS and PMF models are valuable tools for analyzing the sources of heavy metal contaminants in soil. In terms of data input, the PCA/APCS model is less demanding, requiring only concentration data for heavy metals, whereas the PMF model necessitates additional uncertainty data. Operationally, the PCA/APCS model does not rely on specific software and can be implemented using general statistical software, offering a broader scope of application. In contrast, the PMF model requires specialized software for implementation. Regarding the determination of the number of factors, the PCA/APCS model utilizes the principal component analysis (PCA) method to identify the number of factors by simplifying complex data; however, the PMF model does not directly provide a reasonable number of factors and necessitates extensive parameter settings and analysis of simulation effects to ascertain the optimal number of factors [81]. In terms of source contribution calculation, the PCA/APCS model is more time-consuming and may yield negative results [82], while the PMF model is faster in computation, and its non-negative constraints ensure that all source contributions are positive, with graphical outputs available. Overall, the results of source identification from both models are largely consistent, identifying agricultural activities, transportation, and natural parent materials as the main sources of heavy metals in the study area. However, in terms of model fitting and calculating source contribution rates, the Positive Matrix Factorization (PMF) model demonstrates superior performance and yields more reliable results, consistent with findings from previous research [52].

## 4. Conclusion

This study analysed the spatial distribution characteristics and pollution status of soil heavy metals in the planting area of characteristic agricultural products and analysed their sources. The main conclusions obtained are as follows:

1. The concentrations of Cr, Cu, Zn, Pb, Ni and Hg in farmland soils did not exceed the risk screening values of soil pollution in agricultural land, and the overall pollution risk was low.
2. The spatial distribution of Pb and Ni was affected by both natural and human factors, and the spatial distribution of Zn, Cu, Hg and Cr was mainly affected by natural factors. Through Kriging spatial interpolation, the distribution characteristics of Cr, Cu, Zn, Pb and Ni elements are similar, with higher concentrations in the east and north and lower concentrations in the middle. The spatial distribution of Hg element is different, and the concentration is higher in only a few areas.
3. The results of the single pollution evaluation show that there is low pollution of Cr and Ni, no pollution of Cu, Pb, Zn, and Hg. The overall comprehensive pollution is at the warning line level, with mild anthropogenic pollution in the northern construction land and eastern cultivated land.
4. Through the comparison of Positive Matrix Factorization (PMF) and Principal Component Analysis/Absolute Principal Component Scores (PCA / APCS) models, PMF model is superior to PCA / APCS model in the calculation of fitting effect and source contribution rate. Therefore, the results of this study are based on the results of PMF model: 47% of Cr comes from transportation, 42% of Cu, 33% of Pb, 37% of Ni, 41% of Zn comes from agricultural activities, and 73% of Hg comes from natural parent material.

This study holds significant importance for monitoring soil quality in the areas where characteristic agricultural products are grown and offers valuable insights for devising strategies to prevent and control heavy metal pollution. The uniqueness of the study area—characterized by its incomplete administrative boundaries and limited size—poses challenges in collecting data on natural (e.g., soil parent material, slope, soil type, and precipitation) and human factors (e.g., transportation activities, industrial activities, and population density). Consequently, this study did not perform a quantitative analysis of the factors influencing the spatial distribution of soil heavy metals. Future research could benefit from incorporating both local natural and anthropogenic factors to further investigate the spatial distribution of heavy metals.
